# Diabetes Management Experience and the State of Hypoglycemia: National Online Survey Study

**DOI:** 10.2196/17890

**Published:** 2020-06-17

**Authors:** Karim Zahed, Farzan Sasangohar, Ranjana Mehta, Madhav Erraguntla, Khalid Qaraqe

**Affiliations:** 1 Department of Industrial and Systems Engineering Texas A&M University College Station, TX United States; 2 Center for Outcomes Research Houston Methodist Hospital Houston, TX United States; 3 Department of Electrical and Computer Engineering Texas A&M University at Qatar Doha Qatar

**Keywords:** tremor, hypoglycemia, diabetes mellitus, remote sensing technology, survey methods, mobile phone

## Abstract

**Background:**

Hypoglycemia, or low blood sugar levels, in people with diabetes can be a serious life-threatening condition, and serious outcomes can be avoided if low levels of blood sugar are proactively detected. Although technologies exist to detect the onset of hypoglycemia, they are invasive or costly or exhibit a high incidence of false alarms. Tremors are commonly reported symptoms of hypoglycemia and may be used to detect hypoglycemic events, yet their onset is not well researched or understood.

**Objective:**

This study aimed to understand diabetic patients’ perceptions of hypoglycemic tremors, as well as their user experiences with technology to manage diabetes, and expectations from a self-management tool to ultimately inform the design of a noninvasive and cost-effective technology that detects tremors associated with hypoglycemia.

**Methods:**

A cross-sectional internet panel survey was administered to adult patients with type 1 diabetes using the Qualtrics platform in May 2019. The questions focused on 3 main constructs: (1) perceived experiences of hypoglycemia, (2) experiences and expectations about a diabetes management device and mobile app, and (3) beliefs and attitudes regarding intention to use a diabetes management device. The analysis in this paper focuses on the first two constructs. Nonparametric tests were used to analyze the Likert scale data, with a Mann-Whitney *U* test, Kruskal-Wallis test, and Games-Howell post hoc test as applicable, for subgroup comparisons to highlight differences in perceived frequency, severity, and noticeability of hypoglycemic tremors across age, gender, years living with diabetes, and physical activity.

**Results:**

Data from 212 respondents (129 [60.8%] females) revealed statistically significant differences in perceived noticeability of tremors by gender, whereby males noticed their tremors more (*P*<.001), and age, with the older population reporting lower noticeability than the young and middle age groups (*P*<.001). Individuals living longer with diabetes noticed their tremors significantly less than those with diabetes for ≤1 year but not in terms of frequency or severity. Additionally, the majority of our participants (150/212, 70.7%) reported experience with diabetes-monitoring devices.

**Conclusions:**

Our findings support the need for cost-efficient and noninvasive continuous monitoring technologies. Although hypoglycemic tremors were perceived to occur frequently, such tremors were not found to be severe compared with other symptoms such as sweating, which was the highest rated symptom in our study. Using a combination of tremor and galvanic skin response sensors may show promise in detecting the onset of hypoglycemic events.

## Introduction

### Background

Diabetes is a chronic disease affecting more than 9.4% of the world’s population [1], with an estimated US $327 billion in economic costs each year [2]. The majority (about 90%) of the population living with diabetes has type 2 diabetes mellitus (T2DM), while about 10% have type 1 diabetes mellitus (T1DM). Collectively, both types are responsible for around 12% of annual deaths in the United States alone [3]. The management of diabetes is burdensome and requires regular monitoring of blood sugar and careful attention to nutrition.

Fluctuating blood sugar levels outside the normal ranges tend to be common among people with T1DM [4]. Hypoglycemia or low blood glucose (BG) is a dangerous condition that affects people with diabetes when the blood glucose level falls below 70 mg/dL [5]. If the BG level continues to fall below 54 mg/dL, it may result in severe hypoglycemia [5]. Values below this level can cause severe cognitive impairment, seizure, loss of consciousness, and, in some cases, coma [6]. Severe hypoglycemia has also been associated with a higher mortality rate. In one study, 10% of the children surveyed had passed away by the time of follow-up [7]. Over time, recurrent hypoglycemia can inhibit the associated symptoms, leading the affected person to lose sensitivity to or become unaware of hypoglycemic symptoms [6]. When the body is unable to secrete epinephrine that generates hypoglycemic symptoms [8], the risk of death could increase by more than 3-fold [9]. This is particularly risky during sleep where nocturnal hypoglycemia leads to cases of “dead in bed” [10]. Despite evidence suggesting the existence of such self-unawareness and lost sensitivity to hypoglycemic symptoms, little research exists to document the extent of such a phenomenon among patients with diabetes.

The most prevalent technology to monitor BG, particularly for T2DM, is blood glucose meters, which require manual application of a test strip (typically by pricking a finger). The main limitation of traditional meters is that the measurement is periodic and manual. Continuous glucose monitors (CGMs) were commercialized at the beginning of this century [11] and have gained popularity especially among patients with T1DM as they are capable of monitoring BG levels continuously and autonomously. CGMs can provide information about BG trends and can warn against the onset of hyper- and hypoglycemia. However, these tools are invasive and costly and require regular maintenance and calibration [12]. In a large survey of patients with T1DM, around a third of the sample used CGMs [13], and in another survey of 877 CGM users, nearly half noted that they were not satisfied with the cost [14]. More recent studies also showed that CGMs in many cases are not cost-effective [15,16], which generally limits their utility, particularly in medically underserved areas where there is less access to health care [17], less health and technological literacy [18], and, in many cases, a low socioeconomic status. Therefore, there is a critical need to have affordable, noninvasive alternative methods and technologies for monitoring and self-management of diabetes and early detection of hypoglycemic onsets. However, the availability of alternatives, particularly for detection and monitoring of hypoglycemia, has been very limited. A few noninvasive devices such as HypoMon, GlucoWatch G2, and Diabetes Sentry made it to the market but exhibited a high incidence of false alarms and were sensitive to environmental conditions [19]. Those that could not be commercialized were prototypes with significant wearability issues [19]. One study even claimed that noninvasive options were incapable of competing with invasive methods in terms of accuracy [20]. Our overall research objective is to address this gap by designing a noninvasive and cost-effective technology that detects tremors associated with hypoglycemia.

### Objectives

In a previous review, we reported that *tremors* and *trembling* have been found to be very common among patients with diabetes [19]. In another study surveying elderly subjects, trembling was reported in 71% of patients with diabetes [21]. Tremors have been shown to be a significant symptom of hypoglycemia in several other survey studies [22-25] as well as in laboratory studies [26,27]. In this paper, we documented findings from a large survey of patients with T1DM regarding their perception of hypoglycemic symptoms. In particular, we highlighted the differences in how patients perceive the frequency of occurrence, severity, and noticeability of hypoglycemic tremors across age, gender, years living with diabetes, and physical activity to inform the design of future interventions. Additionally, we highlighted patient experiences with technologies used to monitor their blood sugar levels and their preferences for a CGM-alternative wearable device.

## Methods

### Study Design

A cross-sectional internet panel survey of 212 US adults with T1DM was conducted using the Qualtrics platform in May 2019. The study was conducted in accordance with STrengthening the Reporting of OBservational studies in Epidemiology (STROBE) guidelines [28]. After the institutional review board at the authors’ institution reviewed and approved the study protocol, participants were recruited through a Qualtrics panel. Individuals who qualified for the survey based on self-reported demographic data (≥18 years, diagnosed with T1DM) were invited via email to join the panel. The email included information such as the title of the survey, its duration, and a link to follow if they were interested in participating, which would increase their points that can be redeemed later for a reward. To further evaluate this criterion and assess the quality of responses, a pilot data set consisting of the first 10% of responses (n=20) was shared with the research team. Additionally, an automated logic was added to the instrument to automatically remove data that were deemed unreasonable or responses that were not relevant to the question. No identifiable information was recorded, but latitude and longitude were stored by using Qualtrics for each respondent and used to confirm that all participants were located within the United States.

### Survey Design

The survey was designed to target 3 main constructs: (1) perceived experiences of hypoglycemia, (2) experiences and expectations about a diabetes management device and mobile app, and (3) beliefs and attitudes regarding the intention to use a diabetes management device. Questions targeting the first set of constructs attempted to understand the frequency and severity of hypoglycemic tremors when compared with other symptoms of hypoglycemia [29,30]. Additional questions were related to the noticeability of hypoglycemic tremors. These questions were rated by the participants on a 10-point Likert scale (eg, 1=Not Frequent, 5=Neutral, 10=Very Frequent). Questions related to a second set of constructs attempted to document the variety and prevalence of type of technologies such as smartphone apps, CGMs, insulin pumps, and the regular BG meters used for diabetes self-management. Additionally, several questions were designed to elicit patients’ preference for features and characteristics of an ideal diabetes management mobile app and issues related to wearability. Finally, participants were asked about their preference for the frequency of BG measurement and the time of the day in which they preferred such a measurement. Beliefs and attitudes relating to the intention to use a device will be reported elsewhere.

### Analysis

After the pilot data collection and consultation with the research team, a Qualtrics team evaluated the responses for consistency, completeness, and speed of completion. All analyses were performed using JASP (JASP Team, version 0.10.2.). Nonparametric tests were used to analyze the Likert scale data [31]. To compare noticeability, frequency of occurrence, and severity of tremors across genders, a Mann-Whitney U test was performed. To compare them across age groups, years with diabetes, and physical activity, a Kruskal-Wallis test was performed. When a significant difference was found, the analysis was followed with a Games-Howell post hoc test to identify the different groups.

## Results

### Demographics

Participants’ demographics and comparisons with national averages are summarized in Table 1. All participants were located in the United States and represented 40 out of 50 states. Of the 212 participants, 129 (60.9%) were female. A total of 117 participants were between the ages of 30 and 50 years, contributing to more than half the sample size (55.2%). As expected, our data overrepresents the middle age groups and underrepresents older adults who might not be inclined to take a web-based survey. Other demographic factors align with the national data available. A total of 182/212 (82%) individuals in our sample were white non-Hispanic, and 92 participants (43.4%) had a household income greater than US $60,000.

**Table 1:** Participant demographics.

Online data sample			National data		
Characteristics		Values, n (%)	Characteristics	Values, %	References
**Gender**					[32]
	Female	129 (60.9)	—^a^	51.0	
	Male	83 (39.1)	—	49.0	
**Age (years)**					[33]
	18-29	34 (16.0)	20-29	18.4	
	30-39	64 (30.2)	30-39	17.8	
	40-49	53 (25.0)	40-49	16.6	
	50-59	33 (15.6)	50-59	17.4	
	≥60	28 (13.2)	≥60	29.8	
**Race**					[34]
	White	182 (85.9)	—	76.5	
	Native Hawaiian or Other Pacific Islander	2 (0.9)	—	0.2	
	Black or African American	13 (6.1)	—	13.4	
	Asian	6 (2.8)	—	5.9	
	Two or more races	6 (2.8)	—	2.7	
	Other	3 (1.4)	—	—	
	White non-Hispanic	174 (82.1)	—	60.4	
	Hispanic or Latino	17 (8.0)	—	18.3	
**Smartphone**					[35]
	None	15 (7.1)	—	19.0	
	Yes	197 (92.9)	—	81.0	
	Android	103 (52.2)	—	51.1	
	iOS	93 (47.2)	—	48.1	
	Other	1 (0.5)	—	0.8	
**Income level (US $)**					[34]
	<20,000	24 (11.3)	<25,000	19.1	
	20,000 to 29,999	20 (9.4)	25,000 to 35,000	8.8	
	30,000 to 39,999	23 (10.9)	35,000 to 50,000	12.0	
	40,000 to 49,999	17 (8.0)	50,000 to 75,000	17.2	
	50,000 to 59,999	29 (13.7)			
	>60,000	92 (434)	>75,000	42.9	
	Did not answer	7 (33)	Did not answer	—	
**Educational level**					[36]
	Not available	—	None	1.4	
	Less than high school	2 (0.9)	—	4.2	
	High school	36 (17.0)	—	34.9	
	Some college, no degree	43 (20.3)	—	21.0	
	Bachelor's degree	61 (28.8)	—	18.8	
	Associate degree or trade school	20 (9.4)	—	8.2	
	Graduate or professional	50 (236)	—	11.5	
**Years living with diabetes**					
	≤1	69 (32.5)	—	—	
	>1 and ≤10	46 (21.7)	—	—	
	>10 and ≤25	39 (18.4)	—	—	
	>25	58 (27.4)	—	—	
**Daily blood sugar measurements**					
	0	12 (5.9)	—	—	
	1-3	85 (41.7)	—	—	
	4-10	107 (52.5)	—	—	

^a^Not available.

Android users constituted 52.3% (103/197) of smartphone users, and iOS users constituted 47.2% (93/197), while 15 (7.1%) participants indicated that they did not own a smartphone. Participants were also asked how many years they had lived with diabetes. More participants were recently affected (≤1 year; 69/212, 32.5%) or had lived with diabetes for more than 25 years (58/212, 27.4%), compared with >1 year but ≤10 years (46/212, 21.7%), and >10 years but ≤25 years (39/212, 18.4%). Participants were also asked to provide their overall level of physical activity as highly active, active, insufficiently active, or inactive per the guidelines specified by the Office of Disease Prevention and Health Promotion (ODPHP) [37]. The ODPHP definitions were provided as a reference. Of the 212 participants, 50/212 (23.58%) reported to be inactive, 74/212 (34.9%) reported being insufficiently active, 65 (30.6%) participants claimed to be active, and only 23/212 (10.8%) claimed to be highly active. When participants were asked how often they measured their BG level, they reported an average of 3.51 times per day (SD 2.18; range 0-10) with around 97/212 (47.5%) participants performing the measurements less than the required minimum of 4 times a day [38].

### Perception of Hypoglycemic Symptoms

As shown in Table 2, none of the symptoms were rated as very severe or very frequent on average. However, 3 symptoms were reported to be severe (ie, had an average rating above 5). These were sweating, tingly feeling, and change in body temperature. Similarly, 4 symptoms were reported as frequent (sweating, tingly feeling, change in body temperature, and headaches). Severity and frequency were found to be positively correlated using the Spearman rank correlation (ρ>0.8; *P*<.001) for all symptoms listed.

**Table 2:** Average reported rating of severity and frequency of occurrence of different hypoglycemic symptoms.

Symptoms	Frequency^a^		Severity^b^		Spearman correlation, ρ
	Mean (SD)	Median	Mean (SD)	Median	
Nausea	4.15 (2.75)	4	4.08 (2.8)	4	0.88
Change in saliva	4.46 (2.88)	5	4.29 (2.88)	4	0.90
Tremor	4.83 (2.77)	5	4.59 (2.71)	4	0.84
Headache	5.36 (2.92)	6	4.95 (2.97)	5	0.85
Change in body temperature	5.59 (2.87)	6	5.24 (2.89)	5	0.86
Tingly feeling in limbs	5.76 (2.82)	6	5.26 (2.74)	5	0.82
Sweating	5.95 (2.78))	6	5.75 (2.81)	6	0.84

^a^1=extremely rare, 5=neither rare nor frequent, 10=extremely frequent.

^b^1=extremely mild, 5=neither mild nor severe, 10=extremely severe.

Although tremors were generally reported to have medium severity and frequency, when participants were asked how often they encounter hypoglycemic tremors, 110/212 (51.9%) participants reported having hypoglycemic tremors at least once a week (Table 3).

**Table 3:** Reported frequency of occurrence of tremors.

Tremor occurrence	Values, n (%)
Never	11 (5.2)
Rarely	48 (22.6)
Once a month	43 (20.3)
Once a week	36 (17)
Once every few days	39 (18.4)
Once a day	24 (11.3)
More than once a day	11 (5.2)

To compare the effect of hypoglycemia awareness on the perception of symptoms, the question on tremor noticeability was used to split participants into 2 groups. If tremors were rated as less noticeable (≤5), participants were categorized as hypoglycemia impaired; otherwise, they were categorized as hypoglycemia aware. A Mann-Whitney test showed that all symptoms were rated significantly higher in terms of frequency and severity for the hypoglycemia aware group (Table 4).

A separate analysis of variance for tremor noticeability, frequency, and severity was performed to compare differences across gender, age, years with diabetes, and physical activity. A Shapiro-Wilk test confirmed that the data did not adhere to the condition of normality (*P*<.001), possibly because the responses were performed on a 10-point Likert scale.

**Table 4:** Symptom frequency and severity across hypoglycemia impaired or aware groups.

Symptoms	Symptom frequency^a^			Symptom severity^b^		
	Impaired, mean (SD)	Aware, mean (SD)	*P* value^c^	Impaired, mean (SD)	Aware, mean (SD)	*P* value^c^
Nausea	3.08 (2.09)	5.29 (2.93)	<.001	2.87 (1.94)	5.38 (3.01)	<.001
Tremor	3.19 (1.97)	6.59 (2.40)	<.001	2.97 (1.84)	6.33 (2.4)	<.001
Headache	4.49 (2.72)	6.30 (2.84)	<.001	3.99 (2.67)	5.99 (2.93)	<.001
Change in saliva	3.12 (2.23)	5.9 (2.82)	<.001	2.92 (2.24)	5.76 (2.76)	<.001
Sweating	4.87 (2.67)	7.11 (2.41)	<.001	4.46 (2.51)	7.14 (2.44)	<.001
Change in body temperature	4.3 (2.52)	7.0 (2.55)	<.001	3.86 (2.43)	6.73 (2.59)	<.001
Tingly feeling in limbs	4.61 (2.78)	7.01 (2.28)	<.001	4.04 (2.46)	6.57 (2.41)	<.001

^a^1=extremely rare, 5=neither rare nor frequent, 10=extremely frequent.

^b^1=extremely mild, 5=neither mild nor severe, 10=extremely severe.

^c^Mann-Whitney test results.

#### Effects of Gender

First, the noticeability of tremors (dependent variable) was assessed across the 2 genders. A Mann-Whitney test revealed a significant difference (U=3887; *P*<.001), whereby males reported noticing their tremors significantly more than females. In terms of frequency of occurrence, tremors were reported to be higher in males than in females. Males tended to report more tremors once a day, while females reported more tremors *once a month* (Table 5). However, this difference was not statistically significant (U=4661; *P*=.11). The reported severity was significantly different (U=4428; *P*=.03) between females and males (Table 6).

**Table 5:** Frequency of hypoglycemic tremors across genders.

Charecteristics	Female, n (%)	Male, n (%)
Never	6 (5)	5 (6)
Rarely	30 (23)	18 (22)
Once a month	29 (22)	14 (17)
Once a week	23 (18)	13 (16)
Once every few days	25 (19)	14 (17)
Once a day	11 (9)	13 (16)
More than once a day	5 (4)	6 (7)
Total (N)	129	83

**Table 6:** Effect of gender on tremor noticeability, frequency, and severity.

Differences across gender			Participants, n	Median	Mean (SD)	*P* value	
**Noticeability^a^**							
	**Gender**						<.001
		Female	129	5	4.94 (2.55)		
		Male	83	7	6.23 (2.69)		
**Frequency^b^**							
	**Gender**						.11
		Female	129	4	4.57 (2.63)		
		Male	83	5	5.24 (2.95)		
**Severity^c^**							
	**Gender**						.03
		Female	129	4	4.26 (2.61)		
		Male	83	5	5.10 (2.80)		

^a^1=extremely unnoticeable, 5=neither unnoticeable nor noticeable, 10=extremely noticeable.

^b^1=extremely rare, 5=neither rare nor frequent, 10=extremely frequent.

^c^1=extremely mild, 5=neither mild nor severe, 10=extremely severe.

#### Effects of Age

The age groups listed in the demographics were divided into 3 groups. Participants were defined as young if their age was between 18 and 30 years, of middle age if they responded as being aged between 31 and 60 years, and of older age if they responded as being aged ≥60 years. The Kruskal-Wallis test showed a significant difference between the 3 groups (H_2_=14.56; *P*<.001). The older group reported significantly lower noticeability rating compared to both the younger group (median=1.82; SE 0.617; *P*=.01) and middle age group (median=2.166; SE 0.57; *P*<.001). No difference was found between the younger and middle age groups (*P*=.66).

**Figure 1:**
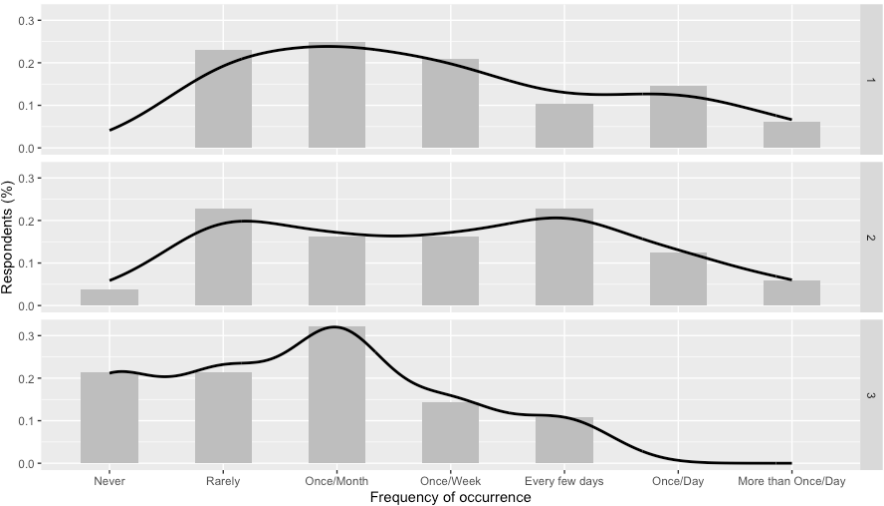
Frequency of hypoglycemic tremors across age groups (top: youngest group [18-30 years]; middle: 30-60 years; bottom: oldest group [≥60 years]).

Differences in the perceived frequency of hypoglycemic tremors were assessed across the 3 age groups. The Kruskal-Wallis test showed no significant difference (H_2_=4.2; *P*=.12) between the younger, middle age, and older groups. However, the older group reported a lower perceived frequency than the other 2 groups, as seen in Figure 1. In particular, the older group did not report any daily tremors; rather, they had a higher number of responses for *once a month* and *never* than the other age groups. A similar analysis was performed for the perceived severity of tremors for the 3 age groups. No significant difference was found (H_2_=5.371; *P*=.07) between the younger group, the middle aged group, and the older group even though the older population tended to perceive the severity of their tremors to be low compared with medium for middle age and young respondents. Table 7 shows a summary of these differences.

**Table 7:** Effect of age on tremor noticeability, frequency, and severity.

Differences across gender			Participants, n	Mean (SD)	Median	*P* value	
**Noticeability^a^**							
	**Age group (years)**						<.001
		18-30	48	5.46 (2.32)	5		
		31-60	136	5.81 (2.64)	6		
		≥60	28	3.64 (2.74)	2.5		
**Frequency^b^**							
	**Age group (years)**						.12
		18-30	48	4.58 (2.583)	4.5		
		31-60	136	5.09 (2.82)	5		
		≥60	28	3.96 (2.76)	3		
**Severity^c^**							
	**Age group (years)**						.07
		18-30	48	4.56 (2.74)	4		
		31-60	136	4.82 (2.71)	5		
		≥60	28	3.54 (2.5)	3		

^a^1=extremely unnoticeable, 5=neither unnoticeable nor noticeable, 10=extremely noticeable.

^b^1=extremely rare, 5=neither rare nor frequent, 10=extremely frequent.

^c^1=extremely mild, 5=neither mild nor severe, 10=extremely severe.

#### Effects of Years With Diabetes

**Figure 2:**
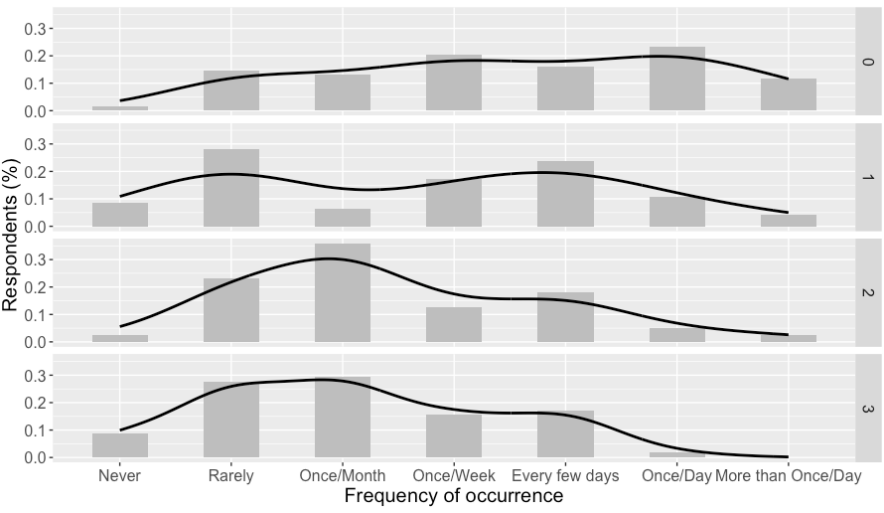
Frequency of hypoglycemic tremors across years with diabetes groups (top: most recently diagnosed; bottom: longest diagnosed).

A significant difference (H_3_=6.322; *P*=.01) between groups was found with regard to the noticeability of hypoglycemic tremors. Those who were more recently diagnosed with diabetes (≤1 year) reported significantly more noticeable tremors (median=1.253; SE 0.479; *P*=.05) than those who had been living with diabetes for more than 25 years (Figure 2).

The effect of years living with diabetes was also analyzed over the frequency of hypoglycemic events, but no significant difference was found (H_3_=5.85; *P*=.12). Similarly, there was no significant difference with regard to the severity of these tremors (H_3_=7.16; *P*=.07; Table 8).

**Table 8:** Effect of years living with diabetes on tremor noticeability, frequency, and severity.

Differences across years with diabetes			Participants, n	Mean (SD)	Median	*P* value
**Noticeability^a^**						
	**Years with diabetes**					.01
		≤1	69	6.03 (2.46)	6	
		>1 and ≤10	46	5.44 (2.61)	6	
		>10 and ≤25	39	5.41 (2.67)	5	
		>25	58	4.78 (2.87)	5	
**Frequency^b^**						
	**Years with diabetes**					.12
		≤1	69	5.44 (2.89)	5	
		>1 and ≤10	46	4.67 (2.65)	5	
		>10 and ≤25	39	4.87 (2.76)	5	
		>25	58	4.21 (2.65)	4	
**Severity^c^**						
	**Years with diabetes**					.07
		≤1	69	5.20 (2.79)	5	
		>1 and ≤10	46	4.59 (2.74)	5	
		>10 and ≤25	39	4.51 (2.50)	4	
		>25	58	3.91 (2.62)	3	

^a^1=extremely unnoticeable, 5=neither unnoticeable nor noticeable, 10=extremely noticeable.

^b^1=extremely rare, 5=neither rare nor frequent, 10=extremely frequent.

^c^1=extremely mild, 5=neither mild nor severe, 10=extremely severe.

#### Effects of Physical Activity

The effect of physical activity levels was assessed with regard to the noticeability, frequency, and severity of hypoglycemic tremors, as summarized in Table 9. For noticeability of hypoglycemic tremors, no significant difference was found between the groups (H_3_=3.98; *P*=.26). Similarly, there was no significant effect of activity level on the perceived frequency of hypoglycemic tremors (H_3_=4.88; *P*=.18) or their perceived severity (H_3_=6.39; *P*=.09).

**Table 9:** Effect of the level of physical activity on tremor noticeability, frequency, and severity.

Differences across levels of physical activity			Participants, n	Mean (SD)	Median		*P* value
**Noticeability^a^**							
	**Level of physical activity**					.26	
		Highly active	23	6.48 (2.94)	7		
		Active	65	5.17 (2.52)	5		
		Insufficiently active	74	5.42 (2.40)	5.5		
		Inactive	50	5.36 (3.06)	5		
**Frequency^b^**							
	**Level of physical activity**					.18	
		Highly active	23	5.78 (3.06)	6		
		Active	65	4.79 (2.70)	4		
		Insufficiently active	74	4.34 (2.50)	5		
		Inactive	50	5.18 (3.04)	5		
**Severity^c^**							
	**Level of physical activity**					.09	
		Highly active	23	5.65 (3.01)	5		
		Active	65	4.75 (2.78)	5		
		Insufficiently active	74	4.00 (2.40)	4		
		Inactive	50	4.76 (2.79)	4.5		

^a^1=extremely unnoticeable, 5=neither unnoticeable nor noticeable, 10=extremely noticeable.

^b^1=extremely rare, 5=neither rare nor frequent, 10=extremely frequent.

^c^1=extremely mild, 5=neither mild nor severe, 10=extremely severe.

#### Technology Preferences

When participants were asked if they had used any technology to manage their diabetes, the majority (150/212, 70.7%) reported that they currently used or had used at least one in the past. Among them, 107/150 (71.3%) used a BG meter, 57/150 (38%) had used a smartphone app, 41/150 (27.3%) had used a CGM, and 49/150 (32.6%) had used an insulin pump to help them with diabetes self-management. Additionally, around 79/150 (52.7%) technology users claimed that they used a combination of these technologies. When asked what device brands they used, the most frequent responses, as listed in Table 10, were *Medtronic, One Touch*, *Dexcom*, *Freestyle Libre*, *Accu-Check*, *Bayer Contour*, *Omnipod*, and *ReliOn*.

**Table 10:** Device brands reported.

Brand	Values, n (%)
Medtronic	25 (16.6)
One Touch	24 (15.9)
Dexcom	17 (11.3)
Freestyle Libre	10 (6.6)
Accu-Chek	7 (4.6)
Bayer Contour	7 (4.6)
OmniPod	7 (4.6)
ReliOn	4 (2.6)
True Metrix	3 (2.0)
Other brands	9 (6.0)
Don't know/unidentified	31 (20.5)

Participants were also asked to rate the important features in an ideal smartphone app that would help them manage hypoglycemia, as commonly found in diabetes management apps [39]. Although all features received favorable ratings, continuous glucose monitoring, insulin log, and graphical display of data received the highest ratings (Table 11).

**Table 11:** Rating of features for a smartphone app to manage diabetes.

Smartphone app features	Mean^a^ (SD)	Median
Glucose monitor	7.11 (2.74)	8
Insulin log	6.59 (2.8)	7
Graphical display of diabetes data	6.55 (2.85)	7
Log for abnormal sugar levels	6.54 (2.9)	7
Food log	6.34 (2.98)	7
Medication log	6.16 (3.01)	7
Reminders	6.14 (3.06)	7
Educational content	5.59 (2.84)	6

^a^1=not important, 5=neutral, 10=very important.

When asked about the characteristics of a diabetes management tool reported in the literature [40,41], high accuracy of readings, low cost, low maintenance, and 24-hour monitoring received very high ratings (Table 12). Other characteristics such as no effects on daily habits, high privacy and security, customizability, and noninvasiveness also received favorable ratings. When asked for their preferred time of the day to measure BG, morning was most preferred (187/212, 88.2%), followed by evening (125/212, 58.9%), night (118/212, 55.6%), afternoon (114/212, 53.8%), and around noon (98/212, 46.2%).

**Table 12:** Rating of characteristics for a device to manage diabetes.

Device characteristics	Mean^a^ (SD)	Median
High accuracy of reading	8.49 (1.88)	9
Low cost	8.21 (2.27)	9
Low maintenance	8.06 (2.18)	9
24-hour monitoring	8.02 (2.28)	9
Doesn’t affect daily habits	7.97 (2.16)	8
High privacy and security	7.85 (2.28)	8
Customizability	7.59 (2.36)	8
Not invasive	7.54 (2.57)	8
Sending health data to caregivers	6.92 (2.62)	7

^a^1=not important, 5=neutral, 10=very important.

A modified Comfort Rating Scale (CRS) [42] was used to evaluate the characteristics of a wearable wrist-worn sensor for hypoglycemia management. Although all constructs related to CRS were rated highly, size and minimized risk for harm received very high ratings followed by emotions felt by the user, social discreteness, and aesthetics (Table 13).

**Table 13:** Rating of items from the comfort rating scale.

Wearability characteristics	Mean^a^ (SD)	Median
Aesthetics (I care about how the device looks)	6.59 (2.85)	7
Social discreteness (I don't want to feel that people look at my wrist and ask about my device)	6.65 (3.01)	7
Emotions (I don't want to feel anxious wearing it)	6.76 (2.95)	7.5
Harm (I don't want this device to cause harm to me)	7.71 (2.67)	9
Size (I want the device to not be bulky)	7.77 (2.34)	8

^a^1=not important, 5=neutral, 10=very important.

## Discussion

A nationwide survey of 212 patients with type 1 diabetes was conducted to investigate noticeability of hypoglycemic tremors as well as perceived frequency and severity of such tremors among patients. Our findings suggest that while tremors are perceived to be less noticeable, frequent, or severe than other hypoglycemic symptoms such as sweating, changes in body temperature, and headache, in line with the literature [19,21], such hypoglycemic tremors occur at moderate frequency and are being noticed by most patients. Indeed, our study shows that more than 50% of the respondents encountered hypoglycemic events at least once a week. This is in line with the established evidence suggesting the rate of one to two mild episodes per week among patients with diabetes [43,44]. Given this prevalence, there is a timely need for the detection and mitigation of mild hypoglycemia before becoming severe [45,46]. However, according to these results, if tremors are tested and found to be a viable predictor of hypoglycemic onset in future work, tremors should be assessed in conjunction with other symptoms as seen in the study by Shechter et al [47]. In past research, relying solely on body temperature and skin conductance was shown to cause a high number of false alarms, which resulted in the devices being withdrawn from the market [48,49].

In addition to these aggregate trends, our findings show gender- and age-specific differences. Although evidence suggests similar occurrence rates of severe hypoglycemia among males and females [50], our findings suggest that males perceive their hypoglycemic tremors more than females. These results are in line with previous findings, which suggest that men were found to have a higher level of adrenaline [51], which is believed to trigger hypoglycemic tremors [52]. In addition, the younger population reported noticing their tremors significantly more than the older population. Similarly, those who had diabetes for a year or less reported noticing their tremors significantly more than those who had diabetes for a longer period. This is in line with previous findings that suggest a radical reduction in the incidence of hypoglycemic symptoms in elderly subjects compared with the younger population [53]. This evidence posits that recurrent hypoglycemia delays the onset of symptoms to lower levels of blood sugar [54] and corroborates previous evidence that patients with a longer history of diabetes may lose sensitivity to hypoglycemic symptoms or perceive such symptoms less [7,8]. These findings further highlight the importance of objective methods for continuous measurement and monitoring of hypoglycemic symptoms in older populations. Participants with higher levels of physical activity also noticed their tremor symptoms more, which may suggest being prone to declining blood sugar levels during and after exercise [55].

While diabetes self-management technologies are gaining popularity, findings from our nationwide survey show that nearly one-third of our sample has not used any technologies to monitor or manage their blood sugar, which suggests low adherence to the basic American Diabetes Association guidelines for the self-management of diabetes [56]. For those who reported using technology, technology adoption was limited to either a blood glucose monitor or a CGM, suggesting the low prevalence of nonintrusive methods for measurement of BG.

As a preliminary step to design a nonintrusive hypoglycemic tremor monitoring tool, we used a patient-centered approach to elicit and document intended users’ preferences and expectations for various features, characteristics, and context of use. It is well understood that incorporating such feedback into the design of patient-facing tools facilitates adoption and increases the odds of sustainable usage [57]. For example, while CGM technologies have proven to be reliable [58], these technologies are not affordable, are invasive, and require frequent maintenance [12,59]. These limitations may explain our survey results, where more than 66% reported not using CGMs. In addition, as evident from our results, for a sensor to be deemed as *wearable* by patients, it should be comfortable, streamlined in appearance, accurate, affordable, and low maintenance. In addition, any smartphone app that connects to the device must provide a graphical display of the patient’s BG data as well as an insulin log. Finally, when participants were asked when they preferred to measure their BG, the most common answers were in the morning and evening, which may suggest expectations for minimal interruptions to professional work. Participants also claimed that they measured their blood sugar approximately four times per day, which is the minimum requirement for T1DM as per several guidelines [38,60]. Although the reported number of measurements ranged from 0 to 10, approximately half of the respondents claimed that they did not check their blood sugar as advised. This bolsters the argument in support of continuous monitoring technologies [61,62], since reliance on users’ memory to sustain usage has proven to be challenging not only for diabetes but also for other chronic diseases [63,64].

Although the study shed light on the nature of perceived hypoglycemic tremor among people with type 1 diabetes and provided information that may guide the design of future tremor-centric interventions, it had some limitations. First, the study only included patients with T1DM, and the results may not generalize to patients with T2DM, especially since hypoglycemia is less common among those patients [65]. In addition, participants were self-identified as T1DM with no objective evidence confirming their condition. Second, the data collected in this study were self-reported. Future work is needed to validate the findings in controlled laboratory environments. Third, since our data were based on Likert scale questions, the analysis was performed using nonparametric tests. However, we believe that our large sample size adds to the robustness of the inference [31]. Finally, a convenience sample was provided using Qualtrics panels. Ideally, a stratified nationwide sample should be used to improve the generalizability of findings.

Regardless of the differences observed in the population studied, this study established the potential efficacy of tremors for a subset of the population as a reliable yet nonintrusive metric for hypoglycemia monitoring technologies and confirms previously reported conclusions [27,47]. The evidence presented in this paper also supports the need for wearable continuous monitoring tools beyond CGMs that are affordable, nonintrusive, and easy to use. Work is in progress to design and evaluate a hypoglycemia monitoring technology that utilizes sensors to detect hypoglycemic tremor and mobile health apps to enable self-management.

## Abbreviations

BG: blood glucose
CRS: Comfort Rating Scale
CGM: continuous glucose monitor
ODPHP: Office of Disease Prevention and Health Promotion
T1DM: type 1 diabetes mellitus
T2DM: type 2 diabetes mellitus
